# The role and function of PPARγ in bladder cancer

**DOI:** 10.7150/jca.42663

**Published:** 2020-04-06

**Authors:** Tianchen Peng, Gang Wang, Songtao Cheng, Yaoyi Xiong, Rui Cao, Kaiyu Qian, Lingao Ju, Xinghuan Wang, Yu Xiao

**Affiliations:** 1 Department of Urology, Zhongnan Hospital of Wuhan University, Wuhan, China; 2 Department of Biological Repositories, Zhongnan Hospital of Wuhan University, Wuhan, China; 3 Human Genetics Resource Preservation Center of Wuhan University, Wuhan, China; 4 Human Genetics Resource Preservation Center of Hubei Province, Wuhan, China; 5 Cancer Precision Diagnosis and Treatment and Translational Medicine Hubei Engineering Research Center, Wuhan, China; 6 Department of Urology, Beijing Friendship Hospital, Capital Medical University, Beijing, China.

**Keywords:** PPARγ, bladder cancer, ROS metabolism, lipid metabolism, chemotherapy sensitivity, ligands

## Abstract

Peroxisome proliferator-activated receptor gamma (PPARγ), a member of the nuclear receptor superfamily, participates in multiple physiological and pathological processes. Extensive studies have revealed the relationship between PPARγ and various tumors. However, the expression and function of PPARγ in bladder cancer seem to be controversial. It has been demonstrated that PPARγ affects the occurrence and progression of bladder cancer by regulating proliferation, apoptosis, metastasis, and reactive oxygen species (ROS) and lipid metabolism, probably through PPARγ-SIRT1 feedback loops, the PI3K-Akt signaling pathway, and the WNT/β-catenin signaling pathway. Considering the frequent relapses after chemotherapy, some researchers have focused on the relationship between PPARγ and chemotherapy sensitivity in bladder cancer. Moreover, the feasibility of PPARγ ligands as potential therapeutic targets for bladder cancer has been uncovered. Taken together, this review summarizes the relevant literature and our findings to explore the complicated role and function of PPARγ in bladder cancer.

## Introduction

Bladder cancer (BCa) is one of the most prevalent malignant tumors in the urinary system 1. Because of a high recurrence rate, bladder cancer patients require frequent follow-up, which is an extremely heavy burden on patients and their families. Approximately 70% of newly diagnosed patients have non‐muscle‐invasive bladder cancer (NMIBC), and 10-20% of patients will progress to muscle‐invasive bladder cancer (MIBC) 2. Diagnosis and treatment review of bladder cancer still relies on traditional diagnosis by cystoscopy biopsy. Furthermore, for patients with NMIBC, the preferred therapeutic method is transurethral resection of the bladder cancer 3. The gold standard treatment for MIBC is radical cystectomy 4. Considering the invasiveness of the diagnostic method and the unfavorable outcomes of the treatment approach, finding new targets for the diagnosis and therapy of bladder cancer has been an urgent problem. Peroxisome proliferator-activated receptors (PPARs), which are members of the nuclear receptor superfamily, can be divided into three subtypes: PPARα, PPARβ and PPARγ 5. Our results and previous studies showed that PPARγ plays a significant role in the occurrence and progression of bladder cancer through regulation of proliferation, apoptosis, metastasis, and reactive oxygen species (ROS) and lipid metabolism 6-10. The purpose of this paper is to provide an overview of the role, function and potential molecular mechanisms of PPARγ in bladder cancer.

## PPARγ and Bladder Cancer

### The expression of PPARγ in bladder cancer

Previous studies have compared bladder cancer with paracancerous tissues, and showed the controversial consequences of the expression of PPARγ in bladder cancer. Cheng et al. searched the Oncomine database and found that there was amplified mRNA expression of PPARγ in BCa tissues 7. In another study, PPARγ expression was evaluated in human BCa and normal bladder tissue samples by FISH assay. The results indicated that 8 out of 21 tumors had PPARγ upregulation while merely 1 out of 23 normal bladder samples showed PPARγ amplification 11. In addition, in a study involving 117 paraffin slice specimens of bladder cancer, researchers found that PPARγ was more likely to be upregulated in the Ta-T1 phase of tumors than in invasive tumors in the T2-T4 phase. Moreover, PPARγ was negatively correlated with the tumor grade, since its expression was higher in tumors of low grade (grade I) than in those of higher grades (grades II and III) 12. In contrast to the above results, Zhang et al. collected information on patients with BCa from The Cancer Genome Atlas (TCGA) and revealed that PPARγ expression was downregulated in BCa 13. Furthermore, in a tissue array of 66 volunteers with BCa, the translation level of PPARγ was significantly increased in paracancerous (normal) tissues 14. Based on the above controversial results, more bladder cancer samples from different races, clinical stages and subtypes are needed to investigate the expression of PPARγ. Regarding cancer prognosis, the results were surprisingly consistent in that amplification of PPARγ mattered greatly in longer survival time and reduced recurrence or progression 12, 14-18.

### The cellular mechanisms of PPARγ functions

**Figure 1:**
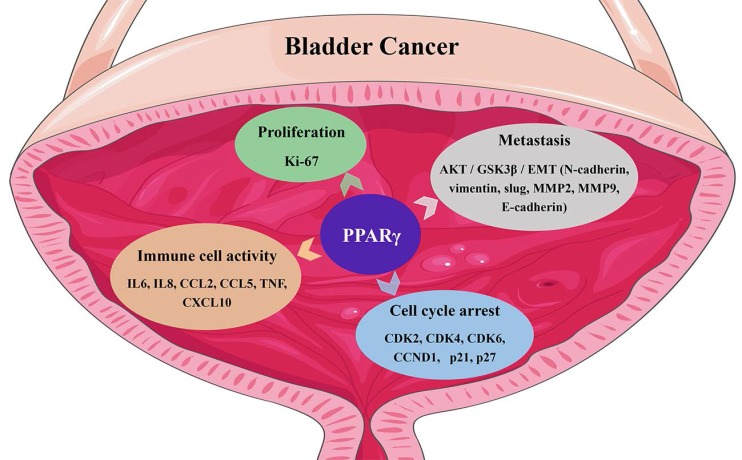
The functin of PPARγ in bladder cancer.

Gene ontology (GO) enrichment analysis of BCa datasets from The Cancer Genome Atlas indicated that PPARγ was related to the regulation of cell proliferation, apoptosis and enhancement of biosynthetic processes 7. Using Kyoto Encyclopedia of Genes and Genomes (KEGG) pathway analysis, PPARγ seemed to be involved in the cell cycle, cell adhesion, cell death, and the PI3K-AKT signaling pathway 7. Moreover, a transcriptome analysis of bladder cancer tissue samples suggested a close correlation between PPARs, sirtuins, cell cycle regulation, ROS metabolism, and the forkhead box class O (FOXO) signaling pathway in BCa 19. PPARγ was reported to display proto-oncogene impacts in metastatic prostate cancer, neuroblastoma and bladder cancer 20, 21. Reducing the expression of PPARγ inhibited bladder cancer cell viability 22, specifically in cell lines expressing a gain or enhancement of PPARγ 21, 23. In addition to its definite function in adipocyte differentiation, PPARγ is also related to the differentiation of many tissues including the urothelium 24. Copy number alterations in PPARγ have also been reported to be associated with luminal tumors 25, 26, which exhibit notable characteristics of strong PPAR pathway activation and amplification of PPARγ and its coactivator and direct transcriptional target, FABP4 27. PPARγ activation also promotes the differentiation of basal bladder cancer cells to the luminal subgroup, cooperating with amplification of GATA3 and FOXA1 or activation of estrogen receptor (ER) 28, 29 and displaying downstream enrichment of CD24, ERBB3, ERBB2, FGFR3, ELF3, CDKN1A and TSC1 mutations and overexpression of E-cadherin, HER2/3, Rab-25 and Src 30, 31. PPARγ also regulates urothelial cell differentiation by inducing the mRNA expression of uroplakin 24. Changes in the cell phenotypes and related gene expression induced by PPARγ in BCa are summarized in Fig. 1.

### The molecular mechanism of PPARγ functions

Ligand binding activates PPARs to translocate to the DNA and bind to retinoid X receptors (RXRs) to form heterodimers and then bind to specific PPAR response elements (PPREs) 32. In turn, this binding promotes gene expression by activating gene transcription. A recent study showed that cell cycle arrest and apoptosis caused by PPARγ agonists may be due to inhibition of the PI3K-Akt signaling pathway 14. PTEN, a nonredundant phosphatase, is of great importance in regulating the PI3K-Akt signaling pathway 33. It has been widely acknowledged that PPARγ inhibits the proliferation, metastasis and invasion of cancer by activating the expression of genes such as PTEN, c-myc and p27 34, 35. Elevated PTEN expression contributes to phosphatidylinositol trisphosphate (PIP3) dephosphorylation, which then suppresses the PIP3 and PI3K/AKT signaling pathways 36-38. PPARγ agonists also upregulate the expression of PTEN 39, resulting in inhibition of the PI3K-Akt signaling pathway in lung cancer 40. Based on the TCGA database, Pearson analysis showed a negative correlation of PTEN and PPARγ expression in BCa 13, which greatly different compared with other cancers. In many pathophysiological states, the activation of PPARγ and its target genes induces inhibition of the WNT/β-catenin pathway 41-43. In nasopharyngeal carcinoma, PPARγ ligands inhibit proliferation and metastasis by regulating E2F2 44. Other signaling pathways including NFκB, EGFR and AP1 are also involved in the molecular regulation of PPARγ 45, 46.

### The interactions of PPARγ with other molecules

**Figure 2:**
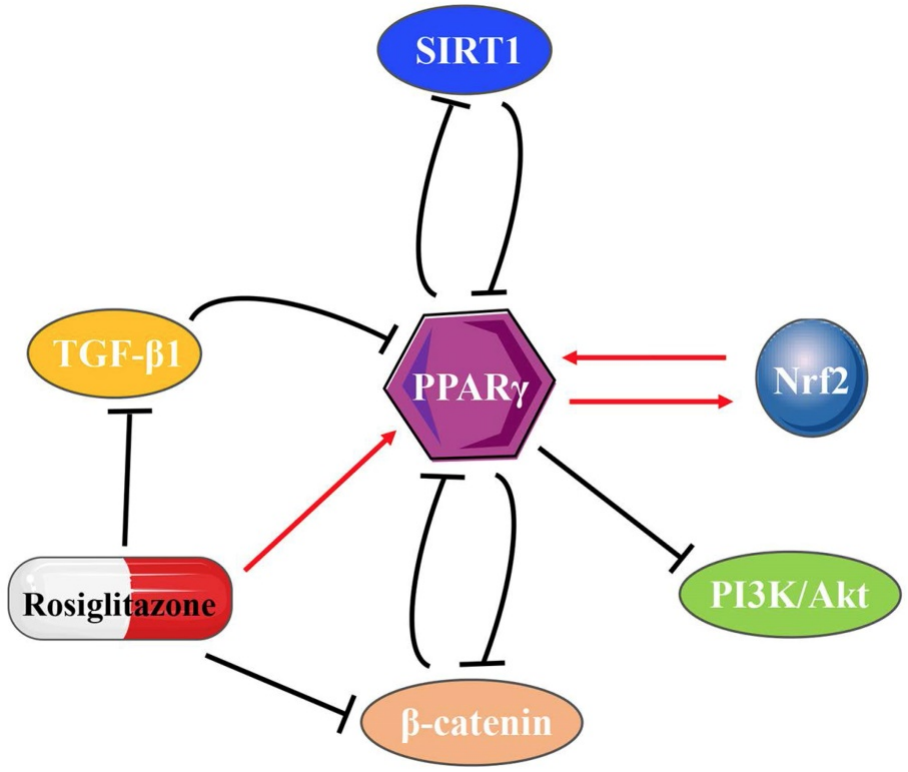
The interactions of PPARγ with other molecules.

PPARγ inhibits SIRT1 at both the transcriptional and translational levels after directly binding to the SIRT1 promoter 47. In turn, SIRT1 also suppresses PPARγ 48, 49, thus forming a negative feedback loop. Transmembrane-4-L-Six-Family-1 (TM4SF1) is a member of the L6 family and strongly upregulated in MIBC. The regulatory ability of the PPARγ-SIRT1 feedback loop explains the tumor promoting effect of TM4SF1 on bladder cancer 8. Holliday junction recognition protein (HJURP) is a centromeric histone chaperone that also acts on the PPARγ-SIRT1 negative feedback loop, impacting on cell proliferation and apoptosis in bladder cancer 9. TGF-β1 upregulation inhibits PPARγ expression 50, 51, whereas PPARγ agonists also directly inhibit TGF-β1 activity and then suppress the expression of α-SMA 52. In various tumors, typical WNT/β-catenin signaling is usually negatively correlated with PPARγ 53. The WNT/β-catenin pathway directly interacts with the catenin-binding domain on PPARγ through the TCF/LEF domain on β-catenin 41. PPARγ agonists also act on the WNT/β-catenin/PI3K/Akt signaling pathway 54, while Akt plays a part in PPARγ regulatory inhibition 55. Different from the negative feedback loops (Fig. 2) mentioned above, reciprocal regulation found between Nrf2 and PPARγ pathways shows its unique significance 56, 57. PPARγ directly regulated by Nrf2 through the newly identified antioxidant response elements (AREs) on its promoter 58. The presence of putative PPREs has also been found in the promoter regions of Nrf2 59, which provides possible direct binding for PPARγ on the Nrf2 promoter to regulate the Nrf2 pathway. In addition, numerous inflammatory cytokines, chemokines, or intracellular signaling pathways, such as TNF-α, interleukin (IL)-1, IL-13, connective tissue growth factor (CTGF), leptin and lysophosphatidic acid (LPA) decrease PPARγ expression in many pathophysiological states 60-62.

### The variant genotype of PPARγ in bladder cancer

As early as 15 years ago, it was shown that the variant genotype of PPARγ indicated a lower recurrence risk among untreated patients with BCa, and MIBC exhibited a significantly higher frequency of variant PPARγ genotypes than NMIBC 63. Subsequent studies revealed that PPARγ activity was enhanced by the P113S mutation, probably because of the mitogen-activated protein kinases (MAP kinases), which inhibit S112 phosphorylation 64. Recently, a study showed that three recurrent mutations, PPARγM280I, PPARγT475M and PPARγI290M, modify the conformation and structural dynamics of the protein, and subsequently affect the ligand binding domain of PPARγ, resulting in a change in protein activity in luminal bladder cancer 65. The PPARγ/RXRα pathway was presumed to be involved in the mechanism of immune escape in bladder cancer, since it was found to be related to the treatment response of NMIBC patients to Bacillus Calmette-Guérin (BCG) 34, 66. Recurrent mutations in RXRα (S242F/Y) occurred in only 5% of MIBCs, whereas these mutations were enriched in the luminal subgroup of MIBC tumors 25, 67. RXRα (S427F/Y) mutations drive the proliferation of urothelial organoids in the absence of tumor suppressor genes and strengthen the interaction between RXRα and PPARγ, activating the PPARγ/RXRα pathway 68, and downregulating the expression of a series of proinflammatory chemokines. Consequently, tumors rebuild the immune microenvironment to resist immune targeted therapy 67, 69.

These results revealed that the point mutation that activated PPARγ or RXRα mutation that acted through PPARγ-dependent pathways 22 provide convincing evidence for identifying PPARγ as a proto-oncogene in bladder cancer and highlight PPARγ as a promising therapeutic target for luminal MIBC 21.

## PPARγ and ROS Metabolism

The role of ROS metabolism in cancer biology is complicated, similar to a double-edged sword, and it regulates both cell survival and apoptosis, depending on the dose, duration, type, or location 70. Nicotinamide adenine dinucleotide phosphate (NADPH) oxidases and mitochondria are two major sources of ROS generation 71, 72. A moderate amount of reactive oxygen species is very important for the development of tumors, while an excessive amount of ROS suppresses tumors by inducing apoptosis 73-75. Activation or deactivation of PPARs both affect genes related to lipid peroxidation, biological metabolism and stress reactions, including ROS 76. PPARγ induces mitochondrial stabilization and protection against oxidative stress 77. 15d-PGJ2, a PPARγ agonist, induces the production of ROS in bladder cancer cells 78. Moreover, the PPARγ-SIRT1 feedback loop has been identified as regulating cell death, the antioxidative response and ROS metabolism 79, 80.

**Figure 3:**
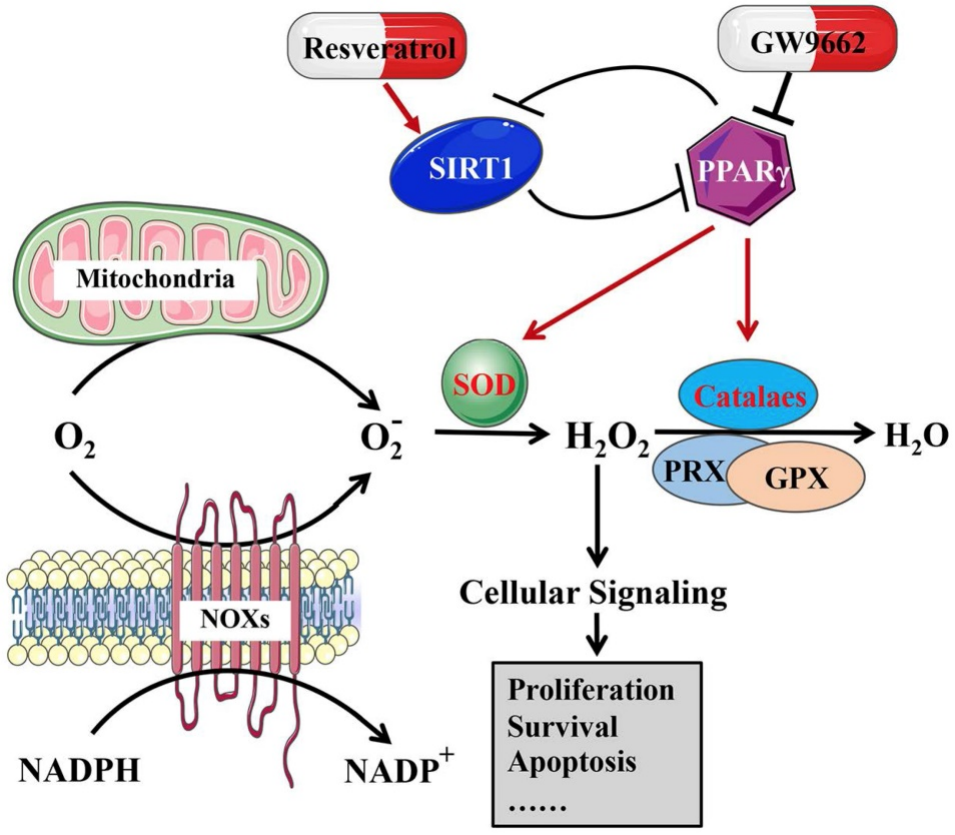
The effect of the PPARγ-SIRT1 feedback loop on ROS metabolism in bladder cancer.

The results of our group revealed that when SIRT1 was knocked down in BCa cells, ROS production was reduced, accompanied by an increase in antioxidant enzymes and total/acetylated FOXO3a, a transcription factor that is the most closely connected to SIRT1 among the FOXO family 19. TM4SF1 is highly expressed in human MIBC. In bladder cancer cell lines, silencing TM4SF1 activated the PPARγ-SIRT1 feedback loop, promoting the formation of ROS and increasing the expression of SOD2 and catalase 8, revealing that excess ROS were produced by disrupting homeostasis, resulting in oxidative stress and death in bladder cancer cells 81. Both PPARγ antagonists (GW9662) and SIRT1 agonists (resveratrol) reduce the excess level of ROS production, which was induced by the knockdown of TM4SF1 in BCa cells 8. In addition, our group found that HJURP could have an impact on the PPARγ-SIRT1 feedback loop, regulating ROS metabolism, and resulting in the progression of bladder cancer 9. Our results, together with previous studies, revealed that the PPARγ-SIRT1 feedback loop mattered greatly in ROS metabolism in bladder cancer (Fig. 3).

## PPARγ and Lipid Metabolism

Reprogramming energy metabolism has been recognized as a hallmark of cancer 82. Otto Warburg first described that cancer cells preferred aerobic glycolysis as the main energy resource even when oxygen was sufficient, which is known as the Warburg effect 83. Recently, many studies have stressed the crucial role of fatty acid oxidation in providing adenosine triphosphate (ATP), NADPH, and other important anabolic materials in cancer cells 84-86. Rather than aerobic glycolysis, in some cancers, lipids have been reported as the primary energy supply 87. As some metabolomics profiling analyses have shown, active fatty acid β-oxidation promotes the development and progression of bladder cancer 88. PPARs are of great importance in the regulation of cell proliferation, cellular differentiation, tumorigenesis and lipid metabolism 89, 90, and can also promote the expression of genes related to fatty acid oxidation and peroxisomal β-oxidation enzymes 91, which are important regulators that maintain the homeostasis of lipid ligands binding to nuclear receptors 92, 93.

**Figure 4:**
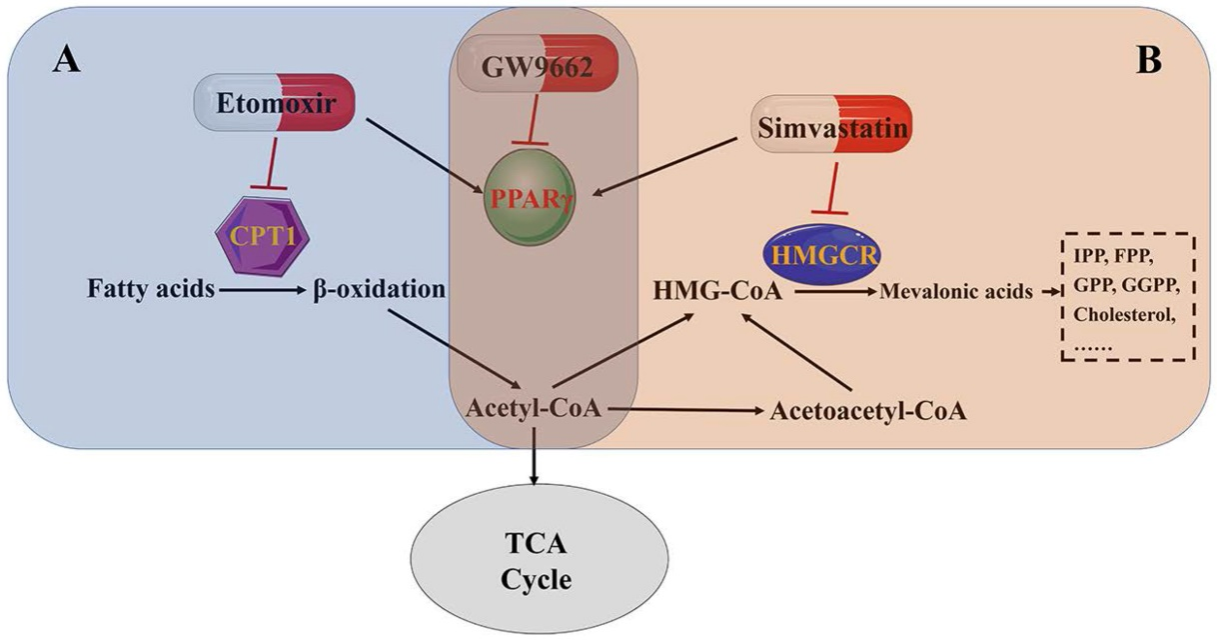
PPARγ affects lipid metabolism in bladder cancer. (A) Fatty acid β-oxidation pathway. (B) Mevalonate pathway.

Our group has performed microarray analysis with human bladder samples. The results revealed that the PPAR signaling pathway mattered greatly in fatty acid/lipid metabolism in bladder cancer 10. Etomoxir, a fatty acid β-oxidation inhibitor, activates PPARγ-mediated signaling, resulting in the cell cycle arrest in BCa cells 6. Moreover, intracellular cholesterol and fatty acids are important components of the cell membrane 94, especially in the membrane domains, which are enriched with lipid rafts and are important in cell proliferation and biological metabolism 95, 96. Simvastatin is a widely used statin drug that is important because of its rate-limiting function in cholesterol synthesis. Results from our group showed that simvastatin inhibited cell viability and induced cell cycle arrest, accompanied by PPARγ activation in BCa cell lines. A PPARγ antagonist (GW9662) restored BCa cells from simvastatin-induced cell cycle arrest 10. These results further demonstrate that lipid metabolism in bladder cancer is regulated by the PPARγ signaling pathway (Fig. 4 A and B).

## PPARγ and Chemotherapy Sensitivity

Of bladder tumors, nearly 70% are low-grade non-muscle invasive cancers, which are not life-threatening but have a distinct tendency to relapse. Maintaining life-long surveillance is very expensive 97. Since current treatments for bladder cancer are so unsatisfactory, new therapy is urgently needed. In American and European consensus guidelines, it is recommended to use intravesical infusion of chemotherapy drugs to inhibit the progression of NMIBC, and BCG is the preferred treatment 98. BCG triggers an immune response, inducing in vitro apoptosis of BCa cells 99-101. Moreover, the function of BCG in inducing the expression of PPARγ in murine BCa has been proven both in vitro and in vivo 34, 66. In a bladder cancer cell line (MB49), BCG induced PPARγ amplification, nuclear translocation and transcriptional activity, resulting in proliferation inhibition, which was strengthened by 15-d-PGJ2, a PPARγ agonist 34. The molecular mechanism might be that intravesical perfusion of BCG stimulates the immune response and induces antitumor activity 102, while PPARγ plays a role in the immune response, since rosiglitazone specifically increases natural killer T-lymphocyte cell-specific receptors 103. In other studies, researchers found that the variant PPARγ allele was associated with a reduced risk of recurrence and progression in BCG-naïve patients and an increased risk of recurrence and progression in those patients who had received prior BCG therapy 63. According to the above results, the combination of PPARγ agonists with certain chemotherapy drugs might be a potential strategy for the treatment of NMIBC 102.

In addition, presurgical (neoadjuvant) cisplatin-based chemotherapy (NAC) is the preferred therapy for high-risk MIBC 104. Treatment selection depends heavily on clinical pathological features, unfortunately current staging systems are woefully inaccurate and result in an increased likelihood of disease progression and eventual death because of inadequate treatment 105. Choi et al. reported that MIBCs can be classified as basal or luminal subtypes. Compared with basal MIBCs, luminal MIBCs are more sensitive to NAC, which can be interpreted as an indication that luminal MIBCs are more likely to have an amplification of PPARγ and activate FGFR3 mutations 31. Although cisplatin-based chemotherapy is only effective in nearly 30% of MIBC patients, effective identification methods for patients who are sensitive to the therapy have not yet been established 104. Therefore, to reach the maximum efficacy in bladder cancer, conventional chemotherapy should be combined with targeted therapies.

## PPARγ Ligands and Bladder Cancer

Many reports have suggested that the regulation of agonists or antagonists on PPARs in cancer cells may be a potential therapeutic strategy for metabolic diseases and cancers, including bladder cancer 46. However, there is heterogeneity in the role of PPARγ ligands in different cancers. It was reported that the PPARγ agonist rosiglitazone activates PI3K-Akt through PPARγ-dependent biosynthesis of VEGF and leptin 106, 107. In another study, PPARγ agonists upregulated PTEN expression and inhibited the PI3K-Akt signaling pathway in lung cancer 39, 40. Therefore, we summarized the previous studies of PPARγ ligands in bladder cancer in Table 1. Most studies showed that PPARγ activation markedly inhibited cell proliferation, induced cell cycle arrest and promoted apoptosis in bladder cancer cells and suppressed tumor growth or metastasis in vivo 108-112. Most of the effect induced by PPARγ activation was recovered by using the PPARγ antagonist GW9662 113. However, GW9662 did not inhibit the action of thiazolidinedione on cell cycle arrest or apoptosis 114, 115 and failed to affect LC3-II accumulation caused by troglitazone 116, revealing that some PPARγ agonists may take effect through PPARγ activation-independent signaling pathways.

Concerns arise when considering PPARγ ligands as potential chemopreventive and therapeutic agents for bladder cancer. Researchers found that PPARγ agonists cause bad results in rats, not only in bladder cancer 117-120 but also in gallbladder and adipose tumors 121. This might be related to the dose used 122. However, an in vitro study showed that pioglitazone usage did not increase the risk of bladder cancer 123. There is a concept that long-term use of PPARγ agonists such as pioglitazone may increase the threat of bladder cancer in patients, while short-term use does not 124, 125. Another theory recommended that PPARγ agonists induce changes in urine composition, particularly an increase in endogenous urinary solids (urolithiasis), which might explain the relationship between urinary bladder tumors and PPARγ agonists 126. Increased urinary sediment, crystals, and/or calculi can lead to necrosis, erosion, and ulceration of urothelial cells, resulting in increased cell proliferation and ultimately cancer formation through epigenetic mechanisms. Some researchers confirmed that several factors affect the formation of endogenous urine solids, including the pH of urine 127, while others obtained the opposite result, denying that changes in urinary parameters are to blame 119, 128. Overall, the mechanism and effect of PPARγ ligands in bladder cancer remain unclear, and basic and clinical research needs to be expanded and deepened in this area.

## Conclusions

PPARγ is a member of the PPAR family and is expressed in a variety of cancers 129. Activation of PPARγ regulates the expression of multiple target genes and inhibits the proliferation or migration of tumor cells 130. However, its expression in bladder cancer is still controversial. Therefore, more bladder cancer tissue samples from different races, ages, tumor stages and grades need to be analyzed to investigate the clinical relevance of the expression of PPARγ in bladder cancer.

Abnormal metabolism is an important feature of malignant tumors 131. Investigating the potential biomarkers or pathways involved in tumor metabolic dysfunction may provide new insights into the diagnosis and treatment of bladder cancer. Our group collected bladder cancer tissue and normal tissue for transcriptomics data analysis. The results revealed that the PPAR signaling pathway is closely related to bladder cancer and may be involved in regulating lipid metabolism 6, 10. Jin et al. reported 12 differential metabolites that contributed to the distinction between the BCa and control groups and many of them were involved in fatty acid β-oxidation 88. Carnitine palmitoyltransferase-1 (CPT1A) is an important rate-limiting enzyme in carnitine-dependent transport across the mitochondrial inner membrane for fatty acid β-oxidation 132. The mRNA expression of CPT1A was significantly higher in MIBC patients than in NMIBC patients 133. In vivo and in vitro experiments by our group showed that etomoxir, a specific inhibitor of CPT1A, inhibited bladder cell proliferation and induced cell cycle arrest in G0/G1 phase by activating the PPARγ signaling pathway 6. In addition, our results also revealed that simvastatin, an inhibitor of cholesterol synthesis, reduced intracellular cholesterol levels and inhibited cell proliferation and migration through the PPARγ signaling pathway 10. The above results confirmed that the PPARγ signaling pathway plays an important role in the regulation of bladder cancer lipid metabolism. However, the precise mechanisms affecting the PPARγ signaling pathway in response to lipid metabolism in bladder cancer remain poorly understood. Increased reactive oxygen species leads to an oxidation/antioxidation imbalance in tumor cells. On the one hand, the accumulation of ROS damages biological macromolecules, leading to a variety of diseases including cancer. On the other hand, it can also selectively kill tumor cells by regulating intracellular ROS levels. Our previous results and related studies showed that the PPARγ can disrupt the homeostasis of ROS metabolism in bladder cancer by affecting the expression of antioxidant enzymes (catalase and SOD2), thereby affecting the occurrence and progression of bladder cancer 8, 9.

Other studies have hypothesized that the PTEN and PI3K-Akt signaling pathways may be important mechanisms for the downstream regulation of PPARγ. Moreover, complex interactions between PPARγ and other molecules, such as negative feedback loops with SIRT1, WNT/β-catenin, and TGF-β1 and reciprocal regulation with Nrf2, also exist in a variety of physiological and pathological conditions, but the specific mechanism still needs to be further investigated.

PPARγ is a ligand-activated transcription factor. Previous studies have shown that PPARγ receptor agonists inhibit the proliferation and growth of tumor cells and enhance the sensitivity of tumor cells to chemotherapies in other malignant tumors. Unfortunately, few studies have investigated the synergistic effects of PPARγ agonists and chemotherapy drugs, such as gemcitabine and docetaxel, in bladder cancer. Taken together, basic research and clinical trials are needed to determine whether PPARγ is a promising biomarker for bladder cancer diagnosis and an effective target for bladder cancer treatment.

## Figures and Tables

**Table 1:** The effect and mechanism of PPARγ ligands in bladder cancer.

Type of Ligands	Cell Type	Type of Effect	Mechanism	Year	Ref.
Rosiglitazone, Pioglitazone	5637, UMUC3	cell growth inhibition, cell cycle arrest, apoptosis induction	inhibition the phosphorylation of Akt (Thr308 and Ser473) and its down-stream molecules including S6, PRAS40 and GSK-3α	2019	14
4-nitrophenol	T24, HUC	promote cellular proliferation, migration and invasion, inhibit adhesion and apoptosis	Bax and E‐cadherin were decreased, N‐cadherin, vimentin, snail, and slug were increased. the expression levels of cancer‐promoting genes HIF‐1, IL‐1β, VEGFα and K‐Ras were enhanced, but p53, PTEN and BRCA were decreased	2019	113
Pioglitazone	NUTE, J82	cell growth inhibition, apoptosis induction	downregulate the protein levels of p53 and cyclin D1	2018	123
15d-PGJ2	T24, 5637	cell growth inhibition, apoptosis induction, sphere formation inhibiton	downregulate the expression of the stemness-related genes, Oct4 and Nanog. facilitate the generation of ROS	2014	78
Troglitazone	T24	induces autophagy, apoptosis, necroptosis	increase the ratio of LC3-II and the phosphorylation of AMPKα. attenuate the phosphorylation of both mTOR and S6K1	2014	116
DIM-Cs	UM-UC1, UM-UC3, UM-UC5, UM-UC6, UM-UC13, RT4、253JP, 253J-BV, KU7	inhibit cell growth and render the resistant cells sensitive to EGFR inhibition	proximal promoters of PPARγ posses CEBP regulatory elements	2013	46
Beta-eleostearic acid (β-ESA)	T24	apoptosis induction	ROS-mediated pathway	2012	108
Ciglitazone	RT4, T24	cell cycle arrest, apoptosis induction	overexpression of p53, p21waf1/CIP1 and p27Kip1. decrease of cyclin B1. increase membrane-bound TRAIL	2011	114
Rosiglitazone, Troglitazone	RT4, T24	cell cycle arrest, apoptosis induction	upregulation of soluble and/or membrane-bound TRAIL. increased cell surface death receptor 5 expression. downregulation of c-FLIP and survivin	2010	115
Telmisartan	T24, Caki-1	cell growth inhibition, apoptosis induction	induce DNA fragmentation	2010	109
Troglitazone, 15d-PGJ2	T24	apoptosis induction	vascular endothelial cell growth factor, mutated p53	2009	110
1,1-bis(3-indolyl)-1-(p-substitutedphenyl) methanes (DIM-Cs)	KU7, 253J-BV	cell growth Inhibition	induced caveolin-1 and p21 expression	2006	111
LY 293111	MCF7, BT 474, SK-BR-3, LL 86, H460,SW480, COLO320/HSR, RT4, HT1197	synergistic to additive effects with gemcitabine in bladder cancer cell lines		2004	112

## Abbreviations

AREs: antioxidant response elements
ATP: adenosine triphosphate
BCa: bladder cancer
BCG: bacillus calmette-guérin
CPT1A: carnitine palmitoyltransferase-1
CTGF: connective tissue growth factor
ER: estrogen receptor
FOXO: forkhead box class O
GO: gene ontology
HJURP: holliday junction recognition protein
IL: interleukin
KEGG: kyoto encyclopedia of genes and genomes
LPA: lysophosphatidic acid
MAP kinases: mitogen-activated protein kinases
MIBC: muscle-invasive bladder cancer
NAC: presurgical (neoadjuvant) cisplatin-based chemotherapy
NADPH: nicotinamide adenine dinucleotide phosphate
NMIBC: non-muscle-invasive bladder cancer
PIP3: phosphatidylinositol trisphosphate
PPARγ: peroxisome proliferator-activated receptor gamma
PPARs: peroxisome proliferator-activated receptors
PPREs: PPAR response elements
ROS: reactive oxygen species
RXRs: retinoid X receptors
TCGA: The Cancer Genome Atlas
TM4SF1: transmembrane-4-L-Six-Family-1
